# Escaping Authenticity’s Dark Side: How Indigenous Groups Negotiate Indigeneity During Contentious Interactions

**DOI:** 10.1177/00076503251384800

**Published:** 2025-11-29

**Authors:** José Carlos Marques, Johnny Boghossian, Diego M. Coraiola

**Affiliations:** 1 University of Ottawa, QC, Canada; 2 Université Laval, Quebec City, QC, Canada; 3 University of Victoria, BC, Canada, and FGV EAESP, Brazil

**Keywords:** authenticity, decolonizing discourse, Indigenous social movement, Indigenous-Settler dialogue, strategic essentialism

## Abstract

Indigenous movement scholarship identifies two primary approaches to claiming indigeneity, strategic essentialism and decolonization, a binary that constrains Indigenous agency by suggesting that Indigenous actors must conform to settler expectations in the short term while postponing decolonization to a later stage. We broaden this perspective by looking at indigeneity from the perspective of constructed authenticity theory, which helps us reveal alternative agentic strategies for claiming identity. We examine how Indigenous leaders, animal rights activists, and policymakers debated Indigenous rights and identity by analyzing claims made during a Canadian summit on fur harvesting. Our findings reveal a clash between non-Indigenous authenticity claims, imposing rigid stereotypes, and Indigenous claims grounded in internal values and self-determination. Polarization persisted until Indigenous leaders reframed authenticity through historical and territorial connections, opening space for dialogue. Our study contributes to Indigenous movement scholarship by showing how different authenticity claims either reinforce settler constraints or foster Indigenous agency.

## Introduction

Scholarship on Indigenous movements highlights the strategic negotiation of externally imposed notions of authenticity as central to resisting colonial structures and asserting self-determination (Coulthard, 2014; Taiaiake, 2005; TallBear, 2013). Scholars have identified two broad strategies: *strategic essentialism*, where Indigenous groups temporarily adopt stereotypical identities to gain political or legal traction (Bell, 2014; Coulthard, 2014; Spivak, 1988), and *decolonizing discourse*, which rejects imposed identities and promotes self-defined narratives and governance (Banerjee, 2021; Peredo, 2023; Tuck & Yang, 2021). While the former offers pragmatic short-term gains within colonial systems, the latter seeks long-term transformation. This binary framing suggests that in direct interactions, Indigenous actors are often constrained to either conforming to settler expectations or openly resisting them.

While considerable research has examined the institutional negotiation of indigeneity, less attention has been paid to how identity claims are contested in face-to-face interactions, particularly in business and political settings. Yet the literature on identity dynamics underscores that such encounters are critical sites where individuals negotiate power, legitimacy, and belonging in real time (Kreiner et al., 2006; Lamont & Molnár, 2002; Langley et al., 2019). These micro-level interactions play a formative role in shaping collective identities, either reinforcing dominant narratives or creating openings for resistance and redefinition. In the Indigenous context, these interactions are shaped by enduring power asymmetries, requiring Indigenous actors to navigate externally imposed authenticity criteria while asserting sovereignty and managing relations with the non-Indigenous. Whether in the context of land negotiations (Pieratos et al., 2021; Tuck & Yang, 2021), reconciliation dialogues (McIvor, 2021), disagreements over pipelines (Estes, 2019), mining disputes (Banerjee, 2000), clashes over fishing rights (Cantzler, 2020; Withers, 2023), or economic development (Henriques et al., 2020; Peredo & McLean, 2013), these interactions reveal how Indigenous authenticity is not only constructed and contested but also strategically mobilized. Studying these encounters offers valuable insight into how identity boundaries are performed, challenged, and reworked beyond formal institutional and legal frameworks.

In this paper, we ask: *How do Indigenous and non-Indigenous groups negotiate “authentic” indigeneity during contentious interactions?* To address this question, we draw on scholarship that conceptualizes authenticity as socially constructed and open for contestation, rather than an objective or fixed attribute (Carroll & Wheaton, 2009; Peterson, 2005). This literature emphasizes that authenticity is shaped through discursive claims grounded in broader cultural and political frameworks, often giving rise to disputes over what is considered genuine or legitimate (Boghossian & David, 2021; DeSoucey, 2010; Voronov et al., 2023), and evaluated and negotiated in ways that reflect conformity to norms, internal consistency, or connections to valued histories and places (Lehman et al., 2019).

Our analysis centers on a pivotal moment in late 20th-century Canada, when Indigenous Peoples pursued self-determination by establishing enterprises grounded in Traditional Ecological Knowledge (TEK) to manage wildlife harvesting. This effort was challenged by a powerful anti-fur campaign led by the animal rights movement. We examine their only documented encounter: an international conference to debate Indigenous rights to harvest fur-bearing wildlife, that brought together Indigenous representatives, animal rights activists, government officials, academics, and fur industry actors.

Findings reveal a conflict shaped by contrasting views of authenticity. Non-Indigenous participants, operating from a conformity-based perspective, upheld rigid stereotypes of pre-modern indigeneity and rejected modern tools or trade as inauthentic. Indigenous leaders, by contrast, emphasized a consistency-based view, grounding authenticity in values, worldviews, and adaptive practices. This mismatch deepened polarization. An opportunity to move beyond the impasse emerged only when Indigenous leaders invoked an alternative understanding of authenticity as connection, highlighting ties to land and heritage. This move changed the grounds of the conversation, resonating with non-Indigenous participants and opening space for dialogue and potential common ground between opposing groups.

We contribute to Indigenous movement scholarship (Banerjee, 2021; Bell, 2014; Coulthard, 2014; Madsen, 2012; McKay, 2019; Peredo, 2023; Spivak, 1988; Taiaiake, 2005; TallBear, 2013; Tuck & Yang, 2021) by showing how consistency-based authenticity claims, intended to express lived values, can inadvertently reinforce settler stereotypes and fixed notions of indigeneity. These claims may be as constraining as strategic essentialism, which conforms to external expectations. We also draw attention to the overlooked potential of connection-based authenticity, grounded in ties to land and history, to ease conflict and foster dialogue. Beyond Indigenous studies, our findings extend constructed authenticity research in management and organization studies, which often highlights authenticity as a source of innovation (Beverland, 2005; Lamertz, 2023; Lehman et al., 2019). Our findings reveal a darker side: dominant groups can impose idealized notions of authenticity to silence, disenfranchise, and stigmatize marginalized communities.

## Literature Review

### Navigating Imposed Identities: Strategic and Decolonial Approaches to Indigeneity

The question of authentic indigeneity has long been a site of contention, negotiation, and resistance within business, management, and broader societal contexts. Historically, authenticity has often been defined externally – by colonial states, legal frameworks, corporations, and non-Indigenous consumers – who impose rigid expectations of what it means to be Indigenous (Bastien et al., 2023; McKay, 2019). These definitions frequently prioritize pre-colonial traditions, communal land ownership, and subsistence economies, where indigeneity is only recognized as authentic if it remains unchanged by modernity (Bell, 2014; Coulthard, 2014). Critics argue that this *essentialist* understanding of indigeneity oversimplifies Indigenous identities, reinforcing stereotypes and denying Indigenous Peoples their right to evolve like any other culture (Durney, 2024; Paradies, 2006; Retzlaff, 2005).

Indigenous communities have endeavored to redefine and take ownership of indigeneity and what is considered authentic by contesting these prevailing narratives and constructs imposed by colonial powers. Scholars have identified two broad strategies: engaging in strategic essentialism and advancing a decolonizing discourse.

Strategic essentialism is a deliberate response by Indigenous communities to systemic power imbalances, involving the selective use of stereotypes to engage settler groups and secure rights and resources (Bell, 2014). As a political strategy, it temporarily downplays internal diversity to foster unity and collective action (Fuss, 1989), using pseudo-essentialism to project a cohesive identity through shared symbols, rhetoric, and stereotypes (Spivak, 1988). For example, Indigenous Peoples may evoke the “Noble Native” stereotype, through traditional dress, music, or ecological knowledge, to attract tourists (Conklin, 1997), build alliances with environmental groups and political parties (Green et al., 2020), or gain media visibility for political and legal aims (Muehlmann, 2009). In doing so, marginalized groups assert political identity, resist domination, and navigate systemic barriers (Wolff, 2007).

The strategic use of the “noble savage” identity by Indigenous communities can be a double-edged sword (Conklin, 1997). While it may yield short-term benefits by appealing to settler views of indigeneity as exotic and unchanging (Conklin, 1997; Durney, 2024; Maddison, 2013; Theodossopoulos, 2013), it also risks reinforcing colonial binaries that judge Indigenous Peoples as either fully authentic or not at all (McKay, 2019; Stillman, 2021). Any deviation from these expectations is often dismissed as inauthentic, leading to the denial of rights and exposure to assimilationist pressures (Green et al., 2020; Maddison, 2013). Conklin (1997), for instance, describes how tourists scrutinized the Kayapo in Brazil for signs of modernity, such as satellite dishes or Western clothing, forcing them to continually defend their identity.

In contrast, *decolonizing discourse* has become a vital approach for Indigenous scholars, leaders, and activists seeking to reclaim indigeneity (Acheraïou, 2011; Banerjee, 2021; Peredo, 2023). This perspective challenges static, pre-contact notions of Indigenous culture and instead highlights the fluid, diverse, and evolving nature of Indigenous identity. Some scholars advocate a relational view, arguing that indigeneity is shaped by specific histories of colonialism and dispossession (Harris & Wasilewski, 2004; Harris et al., 2013). Central to this view is the idea that authentic indigeneity is rooted in community-based knowledge, cultural continuity, and self-identification (Harris et al., 2013).

Decolonizing discourse offers a foundation for reimagining organizational and societal systems by centering sustainability, community well-being, and Indigenization, challenging Western-centric paradigms and fostering resilience and equity (Bastien et al., 2023; Peredo, 2023; Pergelova, et al., 2022; Salmon et al., 2023). Indigeneity anchors Indigenous organizing in principles of relational accountability, collective well-being, and ecological sustainability. This approach resists colonial legacies, revitalizes traditional knowledge systems, and supports autonomy and self-determination (Coulthard, 2014; Peredo, 2023; Phillips, 2010; Salmon et al., 2023). Its dynamic nature enables communities to adapt to modern challenges while preserving cultural continuity. For example, the Aymara and Quechua in Bolivia combine ancestral practices with cooperative models to strengthen solidarity economies (Peredo, 2023).

What constitutes “authentic” indigeneity is, therefore, at the center of Indigenous politics. While Indigenous scholars have deeply explored the meaning of indigeneity, less attention has been given to authenticity as a conceptual tool for analyzing identity claims. Building on recent scholarship, we approach authenticity as a flexible, multifaceted construct with varied meanings and implications (Lehman et al., 2019).

### Constructed and Varied Authenticities

Authenticity is a long-standing object of study in management studies, philosophy, psychology, sociology and cultural studies, and conveys the quality of being “genuine” or “real” (Dutton, 2005; Taylor, 1992; Trilling, 1972; Varga & Guignon, 2023; Williams, 2016). The notion arose with and in response to modernity and industrial progress, today carrying diverse understandings affecting nearly all facets of life (Lehman et al., 2019), down to the products we consume (Kovács et al., 2014), our tastes in art and cultural expression (Lindholm, 2007; Peterson, 1997) to how we experience our careers, potentially impacting our work relationships and career progress (Cha et al., 2019). Despite the diversity of the understandings of authenticity and the scholarly traditions that have considered it, there is general agreement that authenticity is *socially constructed and evaluated*, meaning that (a) its meaning is defined and redefined over time according to different claims, (b) it can be understood from diverse perspectives, each potentially holding a different definition of the ‘genuine’, and (c) the judgment by external audiences may or may not agree with the claims being made.

In other words, authenticity is not an inherent or fixed quality, but rather malleable in nature. It can be constructed and reconstructed through claims about the genuine nature of identities, products, or practices (Beverland, 2005). Recent work has identified three major understandings or approaches to claiming authenticity: conformity, consistency, and connection (Lehman et al., 2019; see Table 1). *Conformity*-based authenticity is often examined in the literature on market categories, where membership is judged by how closely an entity resembles a category prototype (Lehman et al., 2019; Vergne & Wry, 2014). These prototypes serve as cognitive shortcuts that help non-experts make quick judgments based on observable cues (Arjaliès & Durand, 2019; Boghossian & David, 2021). Once institutionalized, prototypes become homogenizing forces, essentially stereotypes. This form of authenticity underlies essentialist frameworks and supports strategic essentialism, where simplified representations are used tactically to advance political or advocacy aims.

**Table 1.** Approaches to Claiming Authenticity.

Claim	Conformity	Consistency	Connection
Description	Aligning with externally recognized standards, stereotypes, or categories	Aligning observable practices with internal values and beliefs, or an inherent “true self”	Demonstrating a link between current practices and a valued, often idealized, past or place.
Underlying assumption	Sameness	Uniqueness	Continuity
Interpretative/evaluative nature	Objective	Subjective	Intersubjective
Correspondence in Indigenous movement literature	Essentialism	Decolonialism	NA

Source: Adapted from Lehman et al. (2019).

*Consistency*-based authenticity emphasizes alignment with an entity’s inner values and beliefs. These claims are inherently subjective, requiring evaluators to interpret whether actions plausibly reflect an intrinsic self. Judgments often hinge on whether the entity is “walking the talk” (Cording et al., 2014), with past behavior serving as a key indicator of consistency over time. Without such context, claims can seem less credible. This form of authenticity aligns with decolonizing discourse, which roots authenticity in internal coherence, relational accountability, and lived experience, affirming self-determination and resisting imposed stereotypes.

*Connection*-based authenticity grounds its claims in a perceived link to a valued time or place. This often involves tying present-day products or practices to founding national myths or idealized pasts that evoke collective identity (DeSoucey, 2010; Newman & Giardina, 2010). Such claims frequently rely on nostalgic imagery that critiques modernity by invoking a lost pre-industrial era (Cashman, 2006; Uzelac, 2010). For example, the concept of *terroir* in wine emphasizes ties between the product and its geographic and cultural origins. As such, Designation of Origin frameworks, such as French Champagne or Italian Parmigiano Reggiano, reinforce authenticity by linking goods to historically rooted places and traditions. However, connection-based authenticity often resides in a fixed understanding of time whereby producers must portray their products as having been produced as they always have, at times relegating their modern production equipment away from public view. Novel products do emerge, but the process necessitates the social construction of a distant past and evocation of a valorized place as their origin point to establish their authenticity (Boghossian & David, 2021), often drawing on nostalgic imagery of an idealized pre-industrial era, appealing through implicit critiques of modernity (Cashman, 2006; Uzelac, 2010).

While authenticity as conformity reflects essentialist claims, and authenticity as consistency aligns with decolonial approaches, the perspective of authenticity as connection remains underexplored in the Indigenous social movements literature. This is important, as connection-based authenticity offers a distinct lens for understanding Indigenous identity claims. As the case below shows, these varied forms of authenticity are central to interpreting Indigenous—non-Indigenous interactions. The ways authenticity is asserted or imposed shape dynamics of recognition, conflict, and resistance, and expose ongoing tensions between externally defined identities and Indigenous self-determination.

## Method

### Data Sources

This study draws from a subset of data on a broader campaign to ban fur imports from Canada, a movement that threatened the livelihoods of Indigenous communities reliant on trapping. Despite the campaign’s impact, animal rights activists largely overlooked Indigenous concerns, and direct interaction between the groups was rare. A notable exception occurred in January 1987, when over 300 participants, including Indigenous and animal rights activists, government officials, academics, and fur industry representatives, gathered in Montreal for a two-day conference titled *A Question of Rights: Northern Wildlife Management and the Anti-Harvest Movement* (CARC, 1989). To our knowledge, it was the only direct engagement between the two sides. Our primary data source is the conference transcript, which includes full panel discussions and summaries of four workshops. Each panel featured three to six speakers, presentations, audience Q&A, and open discussion.

We supplemented this data with documents produced by the Indigenous actors present at the conference (see Table 2). Equivalent documents from the animal rights activists’ side were not found. The absence of historical records in this case is not a limitation; rather, it reinforces our argument that animal rights activists generally ignored the Indigenous question at that point in time. In contrast, the Inuit Circumpolar Conference (ICC), representing Inuit Peoples from Canada, Alaska, and Greenland, had created Indigenous Survival International (ISI) in 1984, precisely to counter the anti-trapping threat created by animal activists. Representatives of both organizations were present at the conference. We relied on internal strategy documents produced by the ICC and the public testimony by ISI members at various public commissions and hearings available in the Library and Archives Canada in Ottawa.

**Table 2.** Description of Data.

Source	Description
Conference transcripts	Full transcripts of the conference entitled A Question of Rights: Northern Wildlife Management and the Anti-Harvest Movement, held in Montreal, 29-30 January 1987 (265 pages).
Royal Commission on Seals and Sealing	Royal Commission launched to investigate the collapse of the Canadian Seal hunt. Transcripts of testimonies and briefs submitted by Indigenous witnesses, including members of Indigenous Survival International (134 pages).
Standing Committee on Indigenous Affairs and Northern Development	House of Commons investigation into the animal-rights movement’s campaign against the Canadian fur industry. Testimonies of members of ISI (61 pages).
Indigenous Survival International and Inuit Circumpolar Conference (ICC) internal documents	Resolutions of the ICC at its annual meetings, reports concerning the establishment of an Inuit Regional Conservation Strategy, as well as a market analysis pertaining to an enhanced role for Indigenous Peoples in the fur industry (285 pages).

### Analytical Approach

The conference is a microcosm of the broader struggle between activists and Indigenous groups. We knew that Indigenous Peoples had been vocal against the activists’ attempts to ban the commerce of fur and the implications such a move would have on Indigenous economies. We also knew the animal activists had not addressed the Indigenous question, focusing instead on a generalized criticism of the fur industry. Therefore, we approached the transcripts of the conference inductively to try to understand the positioning of both groups and how they engaged with one another’s statements. In this sense, although we were aware of the macro-discourses around fur trade, our analysis focused on understanding the micro-discourses (Alvesson & Karreman, 2000) as manifested in the positions taken by both animal activists and Indigenous groups regarding important issues associated with the fur trade and its potential ban.

The analysis proceeded in three stages. First, we identified the speakers, their organizational affiliations and the tone of their comments as favorable or against trapping. Table 3 lists the speakers in the order of first appearance in the transcripts. We categorized the panelists as members of broader social groups such as animal activists, Indigenous communities, academia, and the fur industry. While the fracture lines between Indigenous and animal rights activists were evident, academics were split between the two and fur industry members were in favor of the Indigenous position. When coding the pro-trapping perspective, we focused purely on Indigenous speakers. For the anti-trapping statements, we combined animal rights activists and academics. We labeled these groups ‘Indigenous’ and ‘non-Indigenous’ in our findings.

**Table 3.** List of Speakers and Their Organizational Affiliations.^a^

Speaker code	Name	Position-Organization	Group
1	Marianne Stenbaek	Director, Centre for Northern Studies and Research	Academic
2	Robbie Keith	Canadian Arctic Resources Committee	Indigenous
3	Peter Jacobs	Conference Chairperson, Faculté de l’Aménagement Université de Montréal	Academic
4	Neal Jotham	Coordinator, Humane Trapping Program Canadian Wildlife Service - Government of Canada	Government
5	Thomas Coon	Director, Traditional Pursuits, Grand Council of the Cree, Indigenous Survival International	Indigenous
6	Anne Doncaster	President, National Animal Rights Association; Director, Ark II, Toronto Humane Society	Animal rights activist
7	Peter Ernerk	President, Keewatin Inuit Association	Indigenous
8	Mike Holloway	Alaska Friends of the Earth	Animal rights activist
9	Stephen Best	Vice-Chairman, International Wildlife Coalition	Animal rights activist
10	Alison Lemay	Student	Academic
11	Finn Lynge	Chairman, Inuit Circumpolar Conference, Environmental Commission	Indigenous
12	Vernita Zilys	Director, Rural Alaska Community Action Program Anchorage, Alaska	Indigenous
13	John Grandy	Vice-President, Humane Society of the United States	Animal rights activist
14	Roger Blowes	Ontario Trappers Association	Industry
15	Donald Baillie	Canadian Association for Humane Trapping	Animal rights activist
16	William Payne	Student	Academic
17	Douglas Roseborough	Consultant	Industry
18	Kristina Stockwood	Student, McGill University	Academic
19	Cinday Gilday	Communications Director, Indigenous Survival International	Indigenous
20	Charlie Watt	Senate of Canada Ottawa	Indigenous
21	Paul Okalik	Tungavik Federation of Nunavut	Indigenous
22	Sadie Popovitch-Penny	General Manager, Labrador Inuit Association	Indigenous
23	Peter Usher	Consultant	Consultant
24	Valerius Geist	Professor, Faculty of Environmental Design, University of Calgary	Academic
25	Stephen Hazell	Counsel Canadian Wildlife Federation	Animal rights activist
26	Tom Meredith	McGill University	Academic
27	Cynthia Drummond	Coordinating Director, Canadian Society for the Prevention of Cruelty to Animals	Animal rights activist
28	Del Haylock	Executive Director, Fur Council of Canada	Industry
29	Alan Herscovici	Journalist and Broadcaster, Canadian Broadcasting Corporation	Media
30	Michael O’Sullivan	Canadian Field Representative, World Society for the Protection of Animals	Animal rights activist
31	Dave Monture	Secretary Treasurer, Indigenous Survival International (Canada)	Indigenous
32	Michael Bloomfield	Harmony Foundation	Animal rights activist
33	Mark Gordon	President, Makivik Corporation	Indigenous
34	John Livingston	Professor, Faculty of Environmental Studies, York University	Academic
35	Mark Small	Canadian Sealers Association	Industry
36	Jobee Epoo	Inuit Organization	Indigenous
37	George Wenzel	Professor, Department of Geography McGill University	Academic
38	Greg Michalenko	University of Waterloo	Academic
39	Marietta J. B. Lash	Canadian Association for Humane Trapping	Animal rights activist
40	Terry Fenge	Director of Research, Tungavik Federation of Nunavut	Indigenous
41	Yvon Dubé	Coordonnateur des activités en milieux Amérindien et Inuit Gouvernement du Québec	Government
42	Dy Robb	Department of Renewable Resources, Government of the Northwest Territories	Government
43	Janice S. Henke	Anthropologist	Academic
44	Ron Livingston	Department of Renewable Resources Government of the Northwest Territories	Government
45	Mary Simon	President, Inuit Circumpolar Conference	Indigenous
46	Francoise Patenaude	Biologist, Université du Québec	Academic
47	John Merritt	Executive Director, Canadian Arctic Resources Committee	Indigenous
48	Peter Ittinuar	Native Council of Canada	Indigenous
49	Roger Gruben	Inuvialuit Regional Corporation	Indigenous

a Listed in order of first appearance.

Source: CARC, 1989.

Second, we used an inductive approach with in vivo coding to identify key themes and better understand the issues shaping the debate. One central point of contention was subsistence: animal rights activists equated it with minimal self-use for food and clothing, rejecting any monetization of surplus as commerce. Indigenous participants, by contrast, saw trade as integral to subsistence, enabling the purchase of tools and goods necessary for survival. These contrasting framings extended to other topics, including conceptions of killing versus harvesting, individual versus collective interests, trapping bans versus humane methods, universal versus cultural rights, exploitation versus education in development, and preservation versus conservation.

Third, we found that these tensions reflected deeper disagreements over authentic indigeneity. Activists held a romanticized pre-contact ideal, suggesting Indigenous Peoples must either fully embrace tradition or assimilate to modernity. Indigenous participants, however, emphasized that their identity persisted through evolving values and practices, with self-determination, not a return to the past, as the foundation for adapting to modern realities. Ultimately, the two groups were talking past each other, grounded in incompatible understandings of authenticity.

We then investigated the authenticity claims deployed by both Indigenous and animal rights activists. We focused on how authenticity was claimed, accepted or rejected and situated those claims within the historical, cultural, and political contexts of the debate. We grouped the preliminary codes and further coded the data according to authenticity claims by one side and evaluations of those claims by the other. Once we had mapped all claims, we went back to the literature. We found Lehman et al.’s (2019) typology useful to analyze how both groups argued for authenticity, how their underlying views of authenticity prevented dialogue, and how an alternative view of authenticity could provide a path for building common ground.

### Research Context

The conference occurred during an international anti-trapping campaign targeting the Canadian fur industry, which began in 1983. This campaign followed the successful anti-sealing campaign that had led to the collapse of the Canadian sealing industry the same year (Royal Commission on Seals and Sealing, 1986). Building on prior success, animal rights groups shifted their focus to the Canadian fur industry, targeting the steel-jaw leghold trap and seeking a European import ban, which was expected to similarly devastate the Canadian fur industry (Boghossian & Marques, 2018; Jasper & Poulsen, 1993).

In parallel, the ICC developed a strategy to secure greater decision-making authority over natural resources as a step toward self-government. Based on the 1980 World Conservation Strategy, which introduced the concept of ‘sustainable development’ (IUCN, UNEP & WWF, 1980), the Inuit Regional Conservation Strategy advocated for devolving resource management decisions to Indigenous organizations. These organizations would rely on TEK to manage resources, with any proceeds benefiting their communities. The ICC advocated for self-determination, positioning subsistence hunting, trapping, and fishing as central to Inuit culture and self-government efforts (ICC, 1986). The strategy called for expanded Inuit authority over not only traditional hunting and trapping areas but also the broader “ecological processes” sustaining them. This included influence over projects potentially far beyond traditional territories, such as dam construction, mining, petrochemical exploration, and military flyovers. The plan’s scope was expansive, encompassing lands used for harvesting, migration routes, and adjacent areas vital to sustaining ecosystems (ICC, 1986).

The animal rights campaign against fur-trapping posed a major risk to the livelihoods of many Indigenous communities and their self-determination strategy. The disappearance of the sealing industry in coastal Indigenous communities had already revealed the level of misery caused by losing a major source of income in already poor communities (Royal Commission on Seals and Sealing, 1986). The impact of a collapse of the fur industry would be much more widespread, as there were an estimated 50,000 Indigenous trappers across the North. A collapse in trapping would suddenly greatly reduce the size of the territories that would figure into land claims and seriously risk undermining Indigenous groups’ bid for self-determination.

## Findings

### Indigenous Claims of Authenticity as Consistency

Authenticity as consistency refers to claims associating material and observable practices to an inner self elusive to observers, in order to confer meaning to and legitimate those practices. In our context, this inner self translates to the espoused collective values and beliefs of Indigenous Peoples guiding their hunting and trapping practices. Authenticity as consistency was the type of claim most frequently made by Indigenous speakers, who often began speaking of subsistence from a very personal, emotional perspective. To build these claims, speakers worked hard to communicate their broader worldview, which reflected a discourse of love for and stewardship of the environment upon which they depended for survival. Frequently referring to traditional knowledge, they defined themselves as the “original conservationists” (Speaker#31, p.195), guided by ethical principles and codes informing all interactions with nature.

However, this knowledge was as profound as it was difficult to communicate across cultures and within the setting of the conference. The conference itself was held in a university of a large urban center and conducted in English, which may have complicated the task of Indigenous speakers. Some speakers suggested their inability to express themselves fully in English, as in the case of one who apologetically said that “you will have to bear with me if some of my English words are not pronounced correctly” (Speaker#5, p. 14), while others spoke of the informal ways that they had learned the language. The conference setting itself was far removed from the environment informing the worldview they sought to communicate, potentially exacerbating the gap between the highly abstracted and rationalized perspectives advanced by non-Indigenous attendees and the deeply personal and emotional accounts offered by Indigenous speakers. One Indigenous speaker sought to highlight this cleavage in speaking of the competing understandings of wildlife management: “you could come up with many models for management purposes. It’s only in theory, but not in practice. I think this is where the difference is between our knowledgeable Inuit elders and the scientists and the biologists who are educated strictly from a scientific point of view” (Speaker#20, p.78). Another speaker suggested the same idea, though in a much more evocative manner:

My parents were not taught philosophy; they lived their philosophy. What is taught in your universities bears only a faint resemblance, say as much as a toothpick, honed and refined, bears to the living, rustling tree with its trickles of sap running from its roots 20 feet in the ground to a hundred feet in the sky and sunlight (Speaker#12, p. 35).

What virtually all Indigenous speakers sought to communicate was a deep love for the environment and a strict code informing how they were to interact with it. Yet this code did not dictate a veneration or idealization of nature, but rather a recognition that they too were part of its cycles.

Perhaps precisely because of the difficulty to communicate that code, when speaking of the role of subsistence in their lives, Indigenous speakers’ remarks often remained grounded in their subjective, heartfelt experiences. For example, one speaker vividly described the richness afforded by a subsistence lifestyle:

The day before I came, my husband went hunting with five other guys who were urban Yellowknifers. They went out and shot some caribou and chopped off the heads. But, on my request, my husband went behind and collected all the heads and took all the hearts and fetuses and organs, and threw away the fetuses and all the internal organs. When he returned home, my children helped me clean the caribou. We cooked the heads. I took out the brains. We had a real feast. We had some friends over; I took four bags of delicacies–caribou heads, caribou brain, heart–down to the old-folks’ home for very old women–women who were brought up on the land traditionally and whose husbands have died. To see the emotion of thanksgiving in their eyes, for me, is enough to tell you that, as long as there are native people across this country, hunting and trapping and fishing is going to go on, because it goes beyond the question of money (Speaker#19, p.123).

This statement reveals the underlying values and, importantly, how they are intertwined with the social structure of Indigenous communities. While the speaker does not make generalizations about Indigenous culture and only speaks of her personal life experiences, the richness of her story provides the attentive listener great insight into the respect for elders and the importance of community that are manifested during the hunt and the distribution of its proceeds. Unfortunately, as we argue, these nuances may have been lost on their non-Indigenous interlocutors, for whom the key take-away may have been the idealized image of trapping purely for personal consumption, particularly because the accounts did not address the issue of commercial trapping.

### Non-Indigenous Counterclaims of Authenticity as Conformity

Our findings reveal that non-Indigenous speakers resorted largely to evaluations based on conformity to stereotypes, rarely delving deeper into the meanings conveyed by Indigenous speakers. As stated above, Indigenous speakers had given rich, personal accounts of the importance of hunting and trapping to their communities, while conveying a circular understanding of time and grounding their practices in a harsh but spiritually significant place. However, we found that few of these accounts appeared to even register with non-Indigenous speakers, who consistently dismissed them according to simplified understandings and sharply defined true/false dichotomies, as the following statement indicates:

Many of us support native rights. We support subsistence, legitimate, true subsistence use of animals, but are violently opposed to their entering into the commercial luxury fur trade to satisfy the vanity of people in the United States. . . I think, by and large, most people have concluded that true subsistence is appropriate (Speaker#13, p.38).

As part of this black and white understanding, subsistence could only be considered true if the harvest was used purely for food, clothing, or other traditional pursuits. However, economic trade was not alone in irking non-Indigenous attendees, as seemingly non-traditional hunting and trapping methods also appeared dissonant with their stereotype of indigeneity:

Although it might be argued that the public simply does not understand the ‘need’ for cash to hunt, it is a moot point whether the public will ever accept a definition of ‘subsistence’ that includes the use of snowmobiles, motorboats, and high-powered rifles, or the participation of native people in an international, commercial wildlife industry (Speaker#6, p.21).

In this quotation, the problem is not only the trade in wildlife, but any technological advance beyond the stereotypical image of the traditional native hunter or trapper. These exchanges demonstrate a lack of engagement on the part of non-Indigenous speakers to understand what subsistence genuinely meant to Indigenous Peoples or how it related to their cultures and histories.

The power of such simplified understandings was not only that they could be evoked with little reflection, but that they could also be codified into law. For example, one of the animal-rights activists whose organization had recently been involved in the campaign to ban the sale of sealskins by the Aleut of the Pribilof islands in Alaska explained that “our definitions of subsistence are written pretty clearly and pretty thoughtfully in our Marine Mammal Protection Act. At that time, we found that the people really weren’t quite so interested in subsistence use of seal as they were in quasi-commercial use, which means selling to a fur company in South Carolina. We frankly said, ‘No way . . . You can’t do that’” (Speaker#13, p.44). He later outlined what in his eyes were acceptable for the Aleut: “the natives there can sell the skins, but they can only do it if it’s done as a form of native handicraft. They have to be crafted by native people in traditional ways” (Speaker#13, p.50). Without directly speaking to it, this statement revealed a linear understanding of time where ‘tradition’ was locked in a distant and idealized past prior to the arrival of the Europeans.

To such remarks, an Indigenous speaker who had herself been involved in the defense of the rights of the Aleut retorted by illustrating the contradictions of such views. She said that the Aleut were not only being forced to bury the unused portions of carcasses instead of selling them to earn a modest income, but they now also needed to resort to fashioning handicrafts of little cultural significance, such as cribbage boards, to appeal to the tastes of Westerners. Moreover, none of these campaigns and regulations even touched on the veritable causes of recent declines in seal populations, which she explained were the large numbers of incidental catches by fishing trawlers. In effect, Indigenous Peoples were being bound to live lives circumscribed by the simplistic stereotypes imposed upon them, all the while being dislocated from their traditional hunting grounds as they saw their natural resources depleted by external interests.

### Escalating Tensions and Polarization

The constant dismissal of the authenticity claims made by Indigenous speakers led to escalating tensions. The categorical prototypes discussed in the previous section rested on settler stereotypes of Indigenous cultures and histories, stereotypes that were seldom spoken or explored. However, as the discussions progressed and tensions rose, Indigenous speakers more forcefully demanded explanations of how they were to deal with the extreme poverty in their communities—communities that no longer lived a nomadic life but had long been moved into fixed settlements. It is only when pressed on these matters that animal rights activists spoke directly to the topics of Indigenous cultures and history, only to reveal the stereotypes and prejudices that they had not voiced, all the while claiming to help Indigenous Peoples.

Whereas Indigenous speakers had elaborated claims about the authenticity of subsistence with respect to their cultures, non-Indigenous speakers began dismissing these claims by rejecting the underlying cultures. In the following example, the non-Indigenous speaker dismisses such claims by representing the culture as the cause of the poverty itself:

If the native people decide the fur industry does have a future, and they decide to train their children for a life on the trap-line, they will know that some of these children would have become doctors, lawyers, scientists, and, perhaps, trappers; but it would be a choice. Is it in the best interests of these children and the culture of the native people to encourage them to trap, or to encourage them to get an education so that they can make their own choices in life (Speaker#6, p.21).

In this quotation, the speaker represents the subsistence lifestyle as itself a harm to Indigenous children. Ignoring references to the lifestyle as the source of spiritual nourishment, she imposes Western notions of choice and economic development. When she is later pressed to elaborate on how Indigenous youth are to receive said education, she goes on to say:

I think it is perfectly viable if they want it. The native people receive huge amounts of taxpayer money in this country to live in the North. Some of the use of those funds is entirely up to them. If they want to spend it fighting the protest movement, that is their choice, and it is going to cost, and is costing, a lot of money. If they choose to use it to educate their children, that too is their choice (Speaker#6: 27).

She refers to government transfers to Indigenous communities—a topic non-Indigenous speakers evoked numerous times—suggesting both that the financial transfers are more than adequate to meet community needs and that the only viable path forward is to assimilate into dominant culture. In effect, she transforms the already inadequate transfers into a tool of further oppression by using them to justify rejecting the strategies proposed by Indigenous groups and to avoid discussing Indigenous culture.

As the tensions continued to escalate, Indigenous history was drawn into the debate to further support the attacks on Indigenous culture. An animal rights activist offered a retelling of Indigenous history, recounting how the Europeans had used the fur trade to subjugate Indigenous Peoples:

No longer was each tribe a self-contained and self-supporting unit, but from the Arctic to the prairies and from the Atlantic to the Pacific all alike found themselves inextricably enmeshed in the economic system forced upon them from without. One by one, they ceded their territories to the invaders, and wherever European colonization was proceeding, submitted to confinement on narrow reserves. The needs of the colonists then became their needs also, and in the place of their former self-sufficiency, they were reduced to purchasing most of the necessities of life at the European trading stores. . . Today, native people suffer under the legacy of their involvement with the European fur fashion trade and its debilitating effects on their culture (Speaker#9, pp.141–142).

While he could have used this retelling of history to recognize historical injustices, he instead used it to further support the claim that the culture was at the root of the problem, not part of its solution. In the following quotation, he once again inverts the relationship between the subsistence lifestyle and poverty, by blaming the culture for the poverty.

Low education levels are recognized as a characteristic of native and non-native trappers and are offered as a reason for the promotion of trapping. A more enlightened and productive attitude would be to recognize trapping for what it is: the symptom of a debilitating social disease—functional illiteracy. And, the snake-oil of trapping is not, as some would argue, a treatment (Speaker#9, p.144).

While the conference had begun with a focus on the fur trade, the heated exchanges and increasing polarization appear to have contributed to a retrenchment on all sides. Animal rights activists began voicing opposition to all trade in wildlife by Indigenous Peoples, not just their involvement in the fur industry, and denigrated Indigenous cultures more broadly, even if their remarks were shrouded in statements of support to Indigenous Peoples.

### A Momentary Détente by Translating Connection

Despite the heightening tensions, there was one session that stood out for the cordiality of the interactions. It came during a session where an Indigenous community leader described a new pilot project to hunt caribou and develop the market for caribou meat. Despite the pilot project having all the hallmarks of modernity rejected by animal rights activists—such as the commercial trade in wildlife, the use of snowmobiles, the building of a freezer facility and the launching of a province-wide advertising campaign to generate demand for caribou meat among non-indigenous consumers—this session proved to be the least contentious of the day and no activists voiced concerns over the plans to ramp up production.

What appears unique in the presentation is the lengths to which the speaker went to render the notions of time and connection as perceived by Indigenous Peoples meaningful to non-Indigenous conference attendees. The speaker made concrete statements revealing that it was not in the use of modern tools that the project appeared connected to the past, but in the way those tools were used as connected to the social structure of the community:

Equally attractive as a success variable for the commercial caribou hunt in Labrador is its resemblance to the subsistence hunt, which the Inuit have pursued as an integral component in their seasonal cycle. The Labrador Inuit saw that a hunt that involved going to the country on snowmobile, retrieving the caribou, and eviscerating the animals in the country, as is done in the subsistence hunt, and then bringing the animals to the community for removal of the hide and processing of the meat for shipment to market was entirely in keeping with their traditional resource-use practices. This, in effect, legitimizes the commercial hunt. Without such sanction, the commercial hunt would be intrusive. The Inuit would be reluctant to participate in the venture if it were imposed from the outside and not in harmony with the pattern of resource exploitation familiar to them (Speaker#22, p.62).

This quotation reveals that while the tools may evolve, it is what goes unseen to external audiences that is truly most meaningful to Indigenous understandings of authenticity. Notably, it is the underlying social structure, including the distribution of roles within the community, the decision-making processes and the values and beliefs governing the hunt that remain constant across time. It is precisely in those elements that Indigenous Peoples see a direct connection across time between ‘traditional’ practices and the ‘modern’ trade in wildlife. The explanation reveals that the Indigenous community did not take the project lightly, as it needed to be adapted and molded to gain the community’s approval.

The retelling of the pilot project rendered more meaningful an abstract understanding of time that the general statement of past speakers had failed to do. For Indigenous speakers, the past was ever-present and continuously evolving with each generation. Their focus was not on history, but on memory, transmitted across generations. Their view of tradition was not the rote reproduction of an idealized past, as it might be in Western notions of an “authentic” past brought to life in genuine re-enactments. Instead, it was a living tradition, vested in community elders, who permitted the constant adjustments necessary to respond to the evolving challenges of each generation. This is exemplified in the ‘traditional’ wildlife management system used to govern the hunt, which does not function according to immutable laws passed down across centuries, but on the living memories of present-day elders:

Indigenous management system, based on traditional Labrador Inuit experience, where the “village elders” made rules pertaining to hunting methods, areas, sharing of game, and regulations aimed at maintaining a balance between hunting and the availability of wildlife on which the Inuit depended for their survival (Speaker#22, p.61).

As such, these brief excerpts from the speaker’s rich description of the project reveal the extent to which he sought to convey authenticity as connectedness, in a context where an understanding of time, space, and the past is so vastly different across cultures.

Indigenous speakers did speak extensively of time, but theirs was a very different understanding of time. For them, the past was not seen as a fixed moment in a linear flow; instead, it was grounded in social relationships that spanned across generations, blending the past and the future into an ever-present continuity. This is different to European understandings of authenticity as connection that idealizes a fixed moment in history. Indigenous speakers affirmed their continuous presence on their territories across time, linking past, present and future: “We want to make sure, as I indicated many times, that we have animal food, for the use of future generations of Inuit who live in the Arctic. That’s our ultimate goal in the long run. It’s been our practice for thousands of years, and it will continue to be so for thousands and thousands of years to come” (Speaker#8: 227).

However, Indigenous speakers very rarely sought to legitimize any practices simply because they had been performed for centuries. Their notion of the past was constantly evolving and not static as per European conception. Indigenous notions of time flowed through their community elders, who maintained the traditions of their ancestors. It was through the uninterrupted intergenerational transmission of knowledge that their ties in the present flowed up to the ancient past and down into the future, as suggested by Mary Simon, current Governor General of Canada, but then president of the Inuit Circumpolar Conference:

By strengthening our subsistence economy, Inuit elders and others with proven arctic experience will have greater opportunities to pass on to our youth the special skills and complex understanding of our land and marine areas, and enable our people to continue to live compatibly with our environment. Moreover, our distinct spiritual and cultural values and perspectives will be more likely to survive (Speaker#45, p.214).

Respect for place was also a dominant theme in their discourse, a respect that reflected at once a spiritual attachment and an acknowledgment of the hardships that it could engender. This blend of the two understanding of respect are exemplified in the following quotation by Thomas Coon:

When you are northern, you learn to love the surroundings, the environment, the wildlife, and the human beings that are around you. You learn to respect all that is around you. You learn to share your harvest, and you learn to help each other, because that is the only way you will survive in the North (Speaker#5, p.14).

The North represents such a level of harshness that it forces certain social practices upon communities, all the while stirring a profound sense of love. This mix of place as a source of love and hardship was a common refrain among Indigenous speakers who regularly argued that trade in wildlife was the only source of income in that harsh environment.

While abstract claims about time and place did not seem to register among non-Indigenous attendees throughout the conference, the translation of those understandings into the concrete example of the caribou meat project instantiated the notion of indigeneity as connection and seem to have disclosed a potential path forward for mutual understanding between Indigenous and non-Indigenous participants.

## Discussion

### Synthesis of Findings

The findings reveal a profound disconnect between Indigenous authenticity claims and the evaluations imposed by non-Indigenous actors, highlighting the contested nature of subsistence practices in a politically and culturally charged environment. In our case study, whereas Indigenous speakers claimed *authenticity as consistency* to their cultural values, often voiced through deeply personal accounts, their stories only led non-Indigenous conference attendees to hear those elements that reinforced their stereotypical understandings. The reactions of non-Indigenous speakers and their responses in general aligned with the view of *authenticity as conformity* to stereotypical identities. These identities were rooted in rigid, romanticized, notions of the pre-modern Indigenous person. Hunting and trapping involving modern tools or economic exchange were dismissed as inauthentic, reflecting a simplified, binary understanding of indigeneity. In this binary, hunting and trapping were regarded as “cultural” or “traditional” pursuits divorced from income generating activities, the latter being limited to Western-style careers and economic development. With this as their general understanding of the issues, non-Indigenous speakers only saw Indigenous cultures as the source of the problems afflicting Indigenous Peoples and in no way the solution, fueling polarization between the two camps.

The only exception came in the discussion over the caribou hunt pilot project where the speaker effectively translated Indigenous notions of time and connection to non-Indigenous attendees. Although the project was decidedly the most “modern” of those discussed during the day in terms of its use of technology and its stated goal of market development, the speaker succeeded where others had failed by explaining: (a) the precise elements of the project valued for their continuity with the past, and those regarded as necessary modern adaptations; and (b) the internal debates and negotiations that had preceded the project’s launch. Specifically, whereas for external observers material artifacts represented the most salient and accessible elements upon which to render their authenticity evaluations, the speaker drew attention to the distribution of the roles and responsibilities within the community as the primary determinants of authenticity. Likewise, while non-Indigenous attendees were often skeptical, viewing commercial Indigenous projects as strategic ploys to exploit special rights, the speaker revealed the internal debates and negotiations that external observers would typically not have had access to.

### Contributions to Indigenous Social Movement Studies

Our first contribution extends the Indigenous social movement literature by examining how Indigenous identity is negotiated with non-Indigenous groups in contentious, face-to-face interactions. While most research emphasizes institutional negotiations (e.g., Banerjee, 2021; Bell, 2014; Coulthard, 2014), less attention is paid to how identity claims are contested in direct encounters, particularly in business and political settings (Henriques et al., 2020; Pieratos et al., 2021; Tuck & Yang, 2021). Existing work highlights two broad approaches: short-term strategic essentialism and long-term decolonization (Madsen, 2012; Peredo, 2023; Spivak, 1988; Taiaiake, 2005). In direct interactions, however, only the former is typically feasible, requiring temporary conformity to externally imposed notions of authentic indigeneity (Conklin, 1997; Green et al., 2020; McKay, 2019).

This study contributes to the literature by reframing conflict between Indigenous and non-Indigenous as a struggle over competing conceptions of indigeneity, interpreted through authenticity claims (Carroll & Wheaton, 2009; Lehman et al., 2019;). While prior work has focused on essentialist and decolonial perspectives, linking authenticity to either conformity with external norms (Bell, 2014; Peterson, 2005) or consistency in self-definition (Harris et al., 2013; Coulthard, 2014), we introduce a third, underexplored dimension: authenticity as connection. This view emphasizes relational ties to idealized places or historical periods (Cashman, 2006; DeSoucey, 2010), over fixed cultural markers, offering a more nuanced understanding of how identity claims are negotiated in cross-cultural interactions.

Additionally, our findings show that different types of authenticity claims provoke distinct responses from non-Indigenous actors, influencing both the dynamics and trajectory of conflict. When Indigenous groups assert authenticity as consistency and root their identity in internal values and beliefs, conflict tends to escalate (McKay, 2019; Stillman, 2021). These claims directly challenge settler-imposed frameworks, undermining their legitimacy while offering perspectives that are often difficult for outsiders to interpret or assess (Coulthard, 2014; Harris et al., 2013). As a result, non-Indigenous actors frequently respond defensively, reinforcing opposition and intensifying tensions. In contrast, authenticity as connection tends to prompt more neutral responses, although it is perhaps the most difficult to convey because of different understandings of connection in time and space. Nevertheless, it does not directly challenge external authority and instead emphasizes tangible ties to place and history (Newman & Giardina, 2010; Uzelac, 2010), it provides a shared reference point that is more accessible and less confrontational. This distinction highlights that authenticity claims are not merely rhetorical tools but active mechanisms that shape the course of intergroup conflict and negotiation.

By identifying these patterns, our study contributes to both theoretical and practical debates on Indigenous identity politics. Theoretically, it expands the concept of authenticity beyond the essentialism–decolonization binary (Lehman et al., 2019), offering a more fluid and relational perspective. Practically, it suggests an alternative claim-making strategy that avoids the pitfalls of strategic essentialism while enabling more constructive engagement with non-Indigenous actors. Although scholars have long noted the risks of strategic essentialism (Conklin, 1997; Madsen, 2012;), little attention has been given to alternative approaches suited to face-to-face encounters, particularly those that advance self-determination and support Indigenous leaders in educating and advocating for their communities (Harris et al., 2013; Peredo, 2023). By broadening the repertoire of authenticity claims, our findings offer new pathways for Indigenous organizations to assert their rights and perspectives while challenging rigid identity expectations and fostering greater understanding from non-Indigenous audiences.

### Contributions to Constructed Authenticity Literature

Our findings contribute to the dark side of authenticity literature, demonstrating how authenticity can be used not only as a cultural resource but also as a mechanism of control and marginalization (Conklin, 1997; Durney, 2024; Theodossopoulos, 2013; Zaeendar, forthcoming). While management and organization scholars often present authenticity as a positive tool for institutional change and legitimacy-building (Beverland, 2005; Hatch & Schultz, 2017; Khaire & Wadhwani, 2010; Lamertz et al., 2016; Voronov et al., 2023), our research highlights its oppressive potential when imposed through external evaluations. As Fine (2003) and Ruebottom et al. (2022) show, authenticity claims can be weaponized to confine marginalized groups within rigid, outsider-defined identities, stripping them of agency and adaptability. Our work extends this critique by analyzing how Indigenous Peoples organized against the use of conformity-based authenticity requirements as a tool of settler control (Theodossopoulos, 2013).

We recognize that authenticity is often criticized and rejected by scholars who see it as an instrument of Western domination, settler violence, and a growing source of contention among Indigenous communities (Maddison, 2013; Stillman, 2021). While keeping that in mind, we draw from more recent critical Indigenous scholarship that calls for a better understanding of how Indigenous Peoples make sense of it and leverage authenticity to their own ends (Harris et al., 2013; Verbuyst, Forthcoming). In contrast to authors who see authenticity contests as battlegrounds where marginalized communities are denied the right to shape their own futures (Green et al., 2020; McKay, 2019), our research demonstrates that sometimes marginalized groups can reassert their agency over definitions of authenticity of their collective identities. In so doing, we move away from a dualist view of authenticity as an imposed or resisted category to focus instead on how Indigenous and non-Indigenous groups negotiate notions of authentic indigeneity. For instance, we show how relationality emerges as an important component of Indigenous claims to authenticity in contrast to the rigid set of criteria used by animal activists, and how this novel understanding of authenticity mitigates the activists’ resistance to Indigenous claims. This dynamic is evident in the case of the involvement of Indigenous Peoples in the fur trade in the 1980s. Non-Indigenous activists, while professing support for Indigenous cultures, constrained them to an idealized and static vision of indigeneity, rejecting any deviation as inauthentic. In response, Indigenous Peoples used two strategies. The first was to claim authenticity based on value consistency, a strategy that conflicted with the non-Indigenous understanding that Indigenous Peoples are not the same as they were in the past. The second approach emphasized their evolving connectedness with nature and the place, and how they were adapting to the changes in the environment. They restated their Indigenous identity by describing how the commercial caribou hunting pilot project was grounded in their Indigenous knowledge and practices, their attachment to the land and their co-dependence with the natural world and, at the same time, related to important contemporary challenges involving the survival of Indigenous populations and the development of practices of environmental conservation.

Furthermore, our research challenges the prevailing view of authenticity as merely an external validation process, revealing instead the tensions between external and internal authenticity claims. While MOS often examines how authenticity is strategically leveraged, underprivileged groups are rarely afforded this flexibility when their cultures are frozen in time by outsiders. This pattern is evident in the Lamalera of Indonesia, where necessary technological adaptations to traditional fishing were rejected as inauthentic by external observers, ultimately forcing the community to abandon their innovation (Durney, 2024). Similarly, Indigenous participants in our study found themselves unable to modernize their practices without being accused of betraying their cultural identity, undermining their broader struggle for self-determination. The use of constructs such as authenticity continues to work as a form of epistemicide (Santos, 2015), erasing and subsuming Indigenous views and understandings to the categories provided by Western societies. To this end, we wonder what would have been the outcome of this forum if the Inuit epistemologies were considered equally valid and could be expressed without the constraints imposed by Western constructs and categories. Approaching the question of authenticity from an alternative epistemology founded in Indigenous knowledges such as the Maori tangata whenua (Smith, 2000) and the traditional philosophy of Ubuntu (Ajitoni, 2024) could help us see the political underpinnings of discussions of authenticity (Mccormack, 2011), recognize the different value regimes and limits to what can be considered authentic, and envision alternative perspectives and ways of approaching issues of authentic identities in organization studies. We hope our paper offers an initial direction for future research in this area.

## Conclusion

Our research clarifies how Indigenous Peoples can limit conflict while asserting agency when negotiating self-determination with non-Indigenous groups. We describe how Indigenous groups have leveraged both strategic essentialism arguments and decolonizing discourses when negotiating indigeneity with non-Indigenous groups. Our findings extend the literature by showing that social groups diverge not only in their claims of authenticity but also in their understanding of authenticity. Our case shows how Indigenous groups were able to move beyond the impasse between the conformity views of indigeneity held by animal activists and their initial position of authenticity as consistency by focusing instead on an understanding of authenticity as connection. By redefining how authenticity should be understood, they were able to reassert their authority over their livelihood and reframe their self-determination in non-oppositional terms to the views espoused by animal activists.

While our findings provide important insights, they are based on data collected from a single conference, which limits their generalizability across broader contexts. Future research should explore whether similar dynamics and discursive strategies emerge in other institutional environments and types of conflicts. For example, studies could examine how claims to indigeneity play out in disputes over pipelines, fishing rights, or land governance involving Indigenous Peoples. Additionally, future research should further investigate the role of language and discourse in mediating interactions between Indigenous and non-Indigenous groups and shaping identity politics. In particular, it would be valuable to explore whether the sequence of authenticity claims affects how conflicts unfold. A discourse-based approach could provide deeper insights into how narratives of authenticity are constructed, challenged, and strategically deployed in political contests. Moreover, future research could examine intra-Indigenous debates on authenticity and self-definition. Investigating these dynamics could provide a more nuanced understanding of the differences in disputes within Indigenous groups and between them and non-Indigenous groups. It could also shed light on how intra-Indigenous authenticity claims shape community cohesion, leadership, and strategic mobilization, potentially enabling or constraining broader collective action toward self-determination.

Finally, our findings underscore the need for a more critical understanding of authenticity, particularly within management studies; one that recognizes both its empowering and oppressive dimensions. Future research should further examine how power imbalances shape authenticity claims and how marginalized groups resist externally imposed identities. Investigating how different forms of authenticity (for example, conformity, consistency, connection) are mobilized in political and institutional struggles will provide deeper insight into the contradictions and power dynamics that define contemporary authenticity debates. We call on management and organization scholars to further examine the ideological uses of authenticity and its role in political and social power dynamics.
